# Assessing mouse behaviour throughout the light/dark cycle using automated in-cage analysis tools

**DOI:** 10.1016/j.jneumeth.2017.04.014

**Published:** 2018-04-15

**Authors:** Rasneer S. Bains, Sara Wells, Rowland R. Sillito, J. Douglas Armstrong, Heather L. Cater, Gareth Banks, Patrick M. Nolan

**Affiliations:** a Mary Lyon Centre, MRC Harwell Institute, Harwell Science Campus, Oxfordshire, UK; b Mammalian Genetics Unit, MRC Harwell Institute, Harwell Science Campus, Oxfordshire, UK; c Actual Analytics Ltd., Edinburgh, UK; d School of Informatics, University of Edinburgh, Edinburgh, UK

**Keywords:** Home cage, Welfare, Circadian, Motor function, Refinement, Wheel running

## Abstract

An important factor in reducing variability in mouse test outcomes has been to develop assays that can be used for continuous automated home cage assessment. Our experience has shown that this has been most evidenced in long-term assessment of wheel-running activity in mice. Historically, wheel-running in mice and other rodents have been used as a robust assay to determine, with precision, the inherent period of circadian rhythms in mice. Furthermore, this assay has been instrumental in dissecting the molecular genetic basis of mammalian circadian rhythms. In teasing out the elements of this test that have determined its robustness – automated assessment of an unforced behaviour in the home cage over long time intervals – we and others have been investigating whether similar test apparatus could be used to accurately discriminate differences in distinct behavioural parameters in mice. Firstly, using these systems, we explored behaviours in a number of mouse inbred strains to determine whether we could extract biologically meaningful differences. Secondly, we tested a number of relevant mutant lines to determine how discriminative these parameters were. Our findings show that, when compared to conventional out-of-cage phenotyping, a far deeper understanding of mouse mutant phenotype can be established by monitoring behaviour in the home cage over one or more light:dark cycles.

## Highlights

• Automated assessment of mouse home-cage behaviour is robust and reliable.
• Analysis over multiple light/dark cycles improves ability to classify behaviours.
• Combined RFID and video analysis enables home-cage analysis in group housed animals.

## 1 Introduction

Broad based phenotyping of genetically altered mice employs a battery of tests that, when taken together, provide very useful insights into the influence of the target gene on behaviour (Crawley and Paylor, 1997, Loos et al., 2014). As these tests rely on accurately describing the behavioural outcomes, it is vital that they are well-characterised, robust and replicable (Mandillo et al., 2008, Perrin, 2014). Attempts to implement test batteries have uncovered a number of confounds. Firstly, mouse behaviour is flexible, dynamic and adaptive (Loos et al., 2014, Hong et al., 2015) and is influenced by a variety of genetic and environmental factors such as motivation, interaction with the experimenter, experimental design, test order, testing time and environmental enrichment (Crabbe et al., 1999, Chesler et al., 2002, Gerlai, 2002, Richter et al., 2008, Freund et al., 2013, Hånell and Marklund, 2014, Sittig et al., 2016). Secondly, the interpretation of the results is often subjective and under the variable influence of the investigator (Wahlsten et al., 2003). Despite the implementation of standard operating procedures and robust data analysis methodologies, some sources of variability are unavoidable in traditional phenotyping tests as mice need to be handled by the investigator while they are removed from their home cage and placed in a novel test environment (Crawley, 2008, Mallon et al., 2008, Kilkenny et al., 2010, Hrabě de Angelis et al., 2015). Multiple testing platforms for the same behaviour domain and automated data acquisition goes some way to reduce variability but the presence of the experimenter and novelty of the environment may vary the phenotypic outcome (Crawley, 2008, Bains et al., 2016). As a complement to conventional out-of-cage phenotyping batteries, we review here some developments in home-cage automated phenotyping and include examples where this analysis can enrich phenotype datasets in mouse strains and mutants. In the first instance, we cover automated assessment in a number of conventional phenotyping domains and indicate where these have been insightful in defining behavioural changes throughout a 24 h cycle. Secondly, we consider how developments in automated home-cage monitoring systems are being used to continuously assess multiple biologically-relevant phenotypes over long periods.

## 2 Circadian rhythms and sleep

**Fig. 1:**
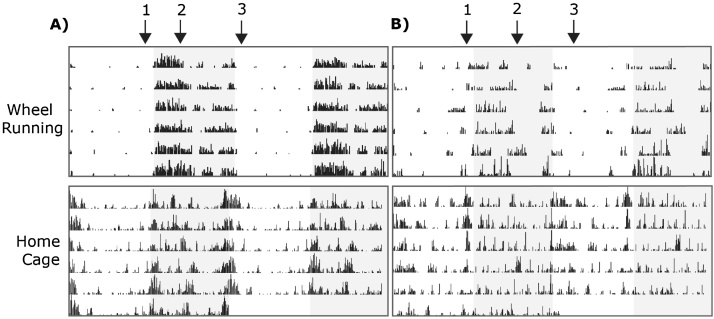
Wheel-running versus home cage activity. Raster plots of total wheel revolutions or total RFID-assessed distance travelled in the home-cage plotted in 6 min time bins over 6 consecutive days in standard 12 h light/dark cycles. The raster plots are double plotted on a 24 h cycle with the shaded area representing the dark phase. Activities of representative animals of two different mouse strains A) C57BL/6J and B) FVB/NcrlBRH. Arrows highlight some of the strain-specific differences in activity that can be distinguished using the two recording systems including 1) differences in anticipatory activity prior to lights-off, 2) abrupt change in activity levels half-way through the dark period and 3) sustained activity following lights-on. Differences between the two systems reflect the fact that wheel-running is an elective behaviour while RFID-based data is collected irrespective of the animal’s voluntary cage activities.

One of the first and widely utilised methodologies used for the long term assessment of animal activity in the home cage is voluntary wheel running. The first reported investigation was published in the late 19th century and used such techniques to analyse the effect of diet and alcohol on the activity of rats (Stewart, 1898). However since then voluntary wheel running has been extensively used primarily to assess circadian rhythms in a variety of rodent species. In such experiments animals are individually housed in cages containing a running wheel and the cages placed in light tight chambers allowing the investigators to modulate the light environment of the animals. Since animals will readily run upon running wheels, the response to changing light environments of the animals can be recorded by monitoring the rotations of the running wheel (see Jud et al., 2005, Banks and Nolan, 2011, Eckel-Mahan and Sassone-Corsi, 2015, for reviews of circadian phenotyping by wheel running). A number of circadian parameters can be assessed in such investigations including the free running circadian period (determined by monitoring activity in constant dark conditions) and the phase of entrainment (determined by monitoring the phase of activity relative to the light:dark cycle). Such parameters have been shown to be sufficiently robust that circadian wheel running can be used to detect differences between different mouse strains (Schwartz and Zimmerman, 1990, Banks et al., 2015), the breakdown of the circadian system with age (Possidente et al., 1995, Banks et al., 2015) or the effect of drug treatment upon the circadian system (Kitanaka et al., 2012). The investigation of activity patterns in inbred strains highlights a number of important features that are time-of-day (phase) dependent (Fig. 1). As a refinement on our insights into the effect of light-dark cycles on rodent behaviour, we find that wheel-running activity shows strain-specific patterns that are not always restricted to the dark (active) phase, nor are they maintained consistently through the dark phase. It is worthy of consideration at this point that all measurable behaviours could display similar strain-specific, time-of-day dependent variation. Consequently, this might warrant a reassessment of how we conduct behavioural screens and specifically whether tests should be carried out at multiple stages throughout the day or even automated to give us a continuous readout of performance across the 24 h cycle. Time-of-day dependent differences in activity might also point to more generalised disturbances in the circadian system. One of the most significant roles circadian wheel running investigations have played is in the identification of the genetic components of the molecular circadian clock. In such experiments genetic ablation or mutagenesis is used in either forward (e.g. identify specific phenotypes and map the causative mutation) or reverse (e.g. disrupt specific genes and assess the phenotype) approaches to identify the key genes and thus the core molecular processes which underpin the cellular clock (e.g. Vitaterna et al., 1994, Bunger et al., 2000, Godinho et al., 2007, Parsons et al., 2015). A key feature of many clock mutants is a disturbance in the phase of activity with onsets in activity being either significantly advanced or delayed relative to the light-dark cycle.

The success and longevity of voluntary wheel running as a method of home cage activity is in part down to its simplicity. Animals are assessed in the home cage, thus removing stress effects of a novel environment which may otherwise affect results and can be assessed for long periods of time to remove intradaily differences in behaviour or activity which may otherwise be present. Additionally, at a practical level the data files produced are small (under 200KB for a month-long screen) and, while analysis can be performed using specialised software, there are some open source options available for analysis of data (e.g. Schmid et al., 2011). Although informative, the presence of the running wheel itself has been shown to impact on a number of behavioural parameters. In general, the introduction of a running wheel to a rodent will lead to an increase in both activity and food intake (Engel et al., 2009, Murray et al., 2010) and furthermore the wheel can have a modulatory effect upon the circadian regulation of metabolism (Pendergast et al., 2014). It has also been noted that certain rodent species will switch from diurnal to nocturnal patterns of activity upon presentation of a running wheel (Blanchong et al., 1999, Kas and Edgar, 1999). Finally an often overlooked aspect of wheel running is its potential to alter behavioural responses of animals. Such behavioural changes include changes in depressive behaviours (Solberg et al., 1999, Bjornebekk et al., 2008) and aggression (Gammie et al., 2003). Notably, wheel running is associated with increased adult neurogenesis (Clark et al., 2010), a process which has been suggested to contribute to behavioural changes (Deng et al., 2010). However, changes in neurogenesis resulting from wheel running have a negligible behavioural effect (Garthe et al., 2016), suggesting that other factors underpin the influence of wheel running on behaviour.

Wheel running activity monitoring is limited to a measure of voluntary activity. This is evident when comparing wheel-running activity records of mouse strains with respective activity measurements using video-tracking based systems (Fig. 1). Although similar patterns of activity are seen, activity measured through a home cage video tracking system is far more detailed and informative and highlights the subtle changes in strain-specific activities over the course of 24 h that are unrelated to the light-dark cycle. Unsurprisingly, wheel-running measurements cannot be used to assess the sleep-wake state of rodents. In order to accurately monitor sleep, traditional approaches have used electroencephalography (EEG) and electromyography (EMG). While such techniques allow high powered analysis of sleep structure and different sleep-wake states, the surgical implantation of electrodes required for such analysis is time consuming and invasive for the animal and therefore is of limited suitability for high throughput or large scale investigations. However recent studies have highlighted that sleep can be assessed in the home cage through activity analysis. Through simultaneous analysis of both EEG and activity, it has been established that episodes of continuous immobility for 40 s or more are very highly correlated with sleep bouts in mice (Pack et al., 2007, Fisher et al., 2012). It is therefore possible to establish high-throughput, non-invasive assessment of sleep in the home cage using activity monitoring systems such as video tracking or infrared beam breaks. While assessment of sleep by immobility does not give the wealth of data that EEG or EMG provides, the high correlation between immobility-defined and EEG-defined sleep means that immobility defined sleep can be used as a behavioural surrogate of sleep with no requirement for invasive surgery. Furthermore since the 40 s epochs of immobility used to track sleep can be defined using commercially available software (e.g. ANYmaze), tracking of sleep by immobility can be implemented with relative ease to any activity monitoring system. Furthermore, using high-quality video recording, it may also be possible to distinguish Rapid Eye Movement (REM) sleep from non-REM sleep using subtle changes in body area and shape (McShane et al., 2012).

Immobility defined sleep screens have been successfully used to identify novel sleep phenotypes in high throughput mouse mutagenesis screens (Potter et al., 2016) and characterise strain and age related changes in sleep (Banks et al., 2015). Furthermore such methodologies have been used to characterise the sleep modulatory effects of the photoreceptor melanopsin (Pilorz et al., 2016, Jagannath et al., 2015), the impact of glutamate receptors on sleep regulation (Pritchett et al., 2015) and characterise sleep in a mouse model of Down syndrome (Heise et al., 2015). It is notable that in the latter study comparisons of activity by video tracking and wheel running demonstrated inconsistencies between the two methodologies such as differences in the peak activity, further demonstrating the impact of wheel running on the activity profile of an animal.

One notable recent addition to the technologies measuring sleep and circadian activity is the COMPASS system (Brown et al., 2016). This technique uses passive infrared monitoring of the home cage to collect activity data over time. The system has been specifically designed to be low cost and open source, eliminating some of the need for specialised equipment and software. Open source analysis tools also allow circadian and immobility defined sleep measures to be taken simultaneously. Since activity is monitored using a passive infrared system, the confounding effects of activity as measured by the running wheel will are removed. While useful in itself, the COMPASS system also demonstrates how sleep analysis by immobility detection can be implemented to activity monitoring and how circadian and sleep analysis can be incorporated to any home cage analysis system with relative ease.

## 3 Motor function

Characterising progressive neurodegenerative and muscle wasting diseases such as Huntington’s disease, Multiple Sclerosis and Duchenne’s muscular dystrophy in mouse models requires a battery of tests to investigate various aspects of motor function and motor coordination (Carter et al., 1999, Liebetanz et al., 2004). Tests such as swimming performance, grip strength measurement and RotaRod assessment are only sensitive to a certain range of motor performance, and have not always been successful in reliably dissecting the differences between wild type and mutant mice (Hara et al., 2001, Liebetanz and Merkler, 2006). In addition, mice can display compensatory behaviour (Carlson and Makiejus, 1990, Hudecki et al., 1993), making the detection of subtle latent motor deficits even harder. Furthermore, these tests are often conducted for a short period of time, in novel environments and in the presence of an investigator, all of which can lead to highly variable results (Crabbe et al., 1999, Wahlsten et al., 2003, Mandillo et al., 2008). In recent years there has been a concerted effort towards automating methods to measure motor function (Wooley et al., 2009, Vandeputte et al., 2010, Chort et al., 2013). Such technologies are aimed at capturing a wider range of behaviours and are free from experimenter bias, but the requirement to remove mice from their home cage into novel environments remains (Scharfers and Claridge-Chang, 2012). One way to overcome these challenges would be to house mice in testing chambers for extended periods of time and measure voluntary activity automatically without interference from the investigator.

To expand the repertoire of meaningful motor function tests in mice, we tested, optimised and validated, a home-cage-based wheel running system to study motor deficits in mice. Standard running wheels such as those discussed in the circadian rhythms and sleep section have shown consistently reproducible results in detecting motor phenotypes in mouse models of Duchenne’s muscular dystrophy and Huntington’s disease (Dupont-Versteegden et al., 1994, Hara et al., 2001, Hickey et al., 2008). By developing this method to introduce complex wheels with missing rungs, latent motor deficits which are central in origin can be detected, including those in mouse models of multiple sclerosis and Parkinson’s disease (Liebetanz and Merkler, 2006, Schalomon and Wahlsten, 2002). To improve on this method further, we developed an automated home-cage-based running wheel system incorporating a conventional wheel with evenly spaced rungs, and a complex wheel, where particular rungs are absent. This apparatus offers a reliable, robust and reproducible test for assessing multiple motor parameters in mice over several weeks in the home cage, as shown by an excellent cross validation across research groups (Mandillo et al., 2014). The system can detect even early onset and/or subtle deficits in motor function consistently in any motor function mutant tested to date. It is particularly encouraging to note that this system has been used to detect motor dysfunctions in two widely-used models of neurodegenerative disease, Huntington’s disease and amyotrophic lateral sclerosis, at ages where other tests (eg. Rotarod) have not yet detected functional deficits. To further improve the discriminative capacity of the test, analysis of data can be focused exclusively on activity during the early night phase, where mice are most active. The latter adaptation can thus be used to focus on the most informative motor parameters in each study.

## 4 Timing and cognition

Given the increased discriminative power of home cage assessments of activity and motor function, we have been interested in establishing whether similar systems could be used to study cognitive function in the home cage. Our goal was to automate and increase the throughput of behavioural testing by combining home-cage behavioural protocols with automated remotely controlled equipment. This was established by restricting access to food in the home cage and introducing a ‘work for food’ paradigm using an operant nose-poke wall (Maggi et al., 2014). A number of successful behavioural paradigms have been included to study interval timing in animals, including peak procedure and switch latency paradigms. The learning and memory component of the task can be assessed by measuring the errors animals make during training. Since the system is included in the home cage it is possible to run the experiment throughout the 24 h cycle rather than restricting trials to particular sessions in the day (Maggi et al., 2014). In this undisturbed home cage task, animals perform self-initiated trails throughout the 24-h cycle and performance is robust and reproducible with low variability in performance. We could collect about 1500 trials per experimental condition using the system and, interestingly, found that the frequency of self-initiated trials was much lower during the light phase and error numbers are greater, the latter presumably related to a sleep inertia effect. In addition, all animals showed an anticipatory burst in nose-poking activity prior to lights-off. The system and test paradigms used were robust enough to identify differences between different mouse strains (Maggi et al., 2014) and could distinguish mouse mutants with different short or long chronotypes in light/dark or constant dark conditions (Balzani et al., 2016). Given this robustness, interval timing is a strong candidate for incorporation into home cage assays which otherwise may not be amenable to cognitive testing.

## 5 Home cage monitoring

Automated analysis of mouse home cage behaviour allows for readily standardised phenotypic experiments to be conducted on a much longer time scale, without the need for human intervention. This means that the data generated is likely to be more reproducible across different laboratories and free from experimenter bias (Wahlsten et al., 2003, Crawley, 2007, Mandillo et al., 2008). Furthermore, investigating perturbations in home cage behaviours allows us to address an entirely new set of questions about mouse behaviour. Behaviours such as grooming, drinking, climbing etc. can be analysed automatically using trainable machine learning algorithms without human intervention for extended periods of time (Jhuang et al., 2010). This is a step change from out of cage methods where conclusions about stereotypic behaviours such as grooming are made based on observations lasting no longer than few minutes (Silverman et al., 2010, Yang et al., 2007).

Automated systems using beam breakers and detectors have been in use for a number of years and are still being used successfully to measure locomotor activity in singly housed mice (Heisler et al., 1998, Tang et al., 2002, Jackson et al., 2003). In their simplest form systems such as the Photobeam Activity System (San Diego Instruments) use the number of beam breaks as a direct indicator of locomotor activity (Heisler et al., 1998). Deeper investigations of these activities enables one to identify patterns that, when fully exploited, can reveal a lot of hidden information about the type of activity on display (Asher et al., 2009). Systems such as LABORAS (Metris B.V.), Activity Monitoring Cage (DiLog instruments Inc.) and PhenoMaster (TSE Systems) have exploited these patterns to build comprehensive ethograms for discrete behaviours such as grooming, climbing, resting and feeding, where a number of events in a set sequence represent a particular behavioural state (Quinn et al., 2003, Goulding et al., 2008, Asher et al., 2009, Edelsbrunner et al., 2009). One of the most quoted examples of this is the work of Goulding et al. (2008), which very elegantly demonstrates the potential of such systems. Here they studied the behavioural components of two lines of obese mice: *ob/ob* (a mouse line lacking the ability to make leptin, an appetite-regulating hormone) and *Htr2c^−/−^* (a mouse line lacking the serotonin receptor 2C that, amongst other functions, influences satiety). They deduced from the animals’ movements that, whilst both mice were obese, the *ob/ob* mice were less active compared to control mice but the *Htr2c^−/−^* indulged in more ‘snacks’ between rest periods. This insight into the nature of genes influencing appetite and satiety would have been overlooked, were it not for continuous monitoring, especially through the dark phase.

Whilst detectors and beam breakers have been used effectively to measure locomotor function and location (Spink et al., 2001, Galsworthy et al., 2005, Morretti et al., 2005), direct visual analysis has expanded our capacity to measure complex home cage behaviours. Video processing algorithms such as Etho Vision XT (Noldus Information Technology), ANY-maze (Stoelting Co) and VideoTrack (ViewPoint Life Sciences Inc), originally used for automatically analysing behaviour tests, can now be combined with their bespoke hardware, PhenoTyper, ANY-maze Cage and PhenoRack, to monitor and categorise home cage behaviours (Salem et al., 2015). In addition, it is also possible to multiplex these systems with a variety of other instruments, such as operant conditioning modules and optogenetic stimulus devices, to investigate specific behavioural traits like memory and anxiety over extended periods of time.

Recent advances in informatics and image analysis have enabled the collection of data with such precision and granularity that systems such as HomeCageScan (CleverSys Inc) are able to capture and categorise over two dozen behaviours including fine movements such as grooming, head bobbing and sniffing (Baker, 2011, Scharfers and Claridge-Chang, 2012). Such detailed information about mouse movement and behaviour has greatly improved our capacity to monitor ill health in mice. In a study by Roughan et al. (2009), postoperative behaviour of two strains of mice was recorded and analysed both manually and using automated behaviour recognition software (HomeCageScan) for changes in response to analgesics. They reported that not only was the system capable of identifying behaviours indicative of pain, but it was also able to detect behavioural changes to increasing doses of analgesia. Continuous monitoring of mice for a prolonged period of time offers a distinct advantage over the existing system where the mice are observed manually for a limited period of time post surgery. Mice are crepuscular animals, which means that they are most active during the dawn and dusk periods, therefore the times when they are most likely to show signs of ill health go unobserved (Richardson, 2015). This concept has been developed further using the HCA home-cage monitoring system (see Fig. 3 and associated text below), where continuous monitoring of group housed mice in the true home cage has shown some hitherto unobserved activity phenotypes in neurodegenerative mutants during the dark phase. Consequently, in combination with regular monitoring by experienced staff, automation can greatly refine post-operative care and welfare monitoring in mice.

## 6 Automated assessment of individual behaviours in group-housed conditions

Mice are social animals in the wild, however, the methods described above require solitary housing. Removing the mouse from its cage-mates and placing it into a novel environment has been shown to affect behaviour, general wellbeing and metabolism, and so could in itself cause behavioural artefacts (Bartolomucci et al., 2003, Sun et al., 2014). Recent advances in computer vision and processing have made it possible to combine inputs from multiple sources to complement each other and generate a much richer data set, where data from trackers and detectors can inform the visual analysis algorithms to create a three dimensional image of the arena with high spatiotemporal precision. This means that it is possible to track multiple animals and quantify complex social interactions free from experimenter bias (Spink et al., 2001, Branson and Belongie, 2005, Galsworthy et al., 2005, Winter and Schaefers, 2011, Ohayon et al., 2013, Shemesh et al., 2013, Weissbrod et al., 2013, Hong et al., 2015, Salem et al., 2015, Bains et al., 2016).

Social interactions are critical to the survival and reproduction of most animal species while a number of human conditions such as Autism Spectrum Disorders (ASD) and schizophrenia have a large social element (Hong et al., 2015). The majority of established automated visual tracking systems are used to study social interactions in pairs of animals in a laboratory environment (de Chaumont et al., 2012, Ohayon et al., 2013, Hong et al., 2015). Such interactions have been invaluable in investigating the neural systems underlying decision making and learning in mice and mouse models of psychiatric conditions such as depression, ASD and aggression (Silverman et al., 2010, de Chaumont et al., 2012). However complex social behaviours are often harder to quantify; the current challenge lies in the ability of visual systems to distinguish between animals and patterns when the mice are in close proximity to each other (Hong et al., 2015) and, in the home cage, this is further complicated by enrichment and nesting materials (de Chaumont et al., 2012, Weissbrod et al., 2013, Hong et al., 2015). A number of systems have overcome this problem of occlusion by using overhead cameras (Shemesh et al., 2013, Patel et al., 2014). The top down view offered by the overhead cameras is more tolerant of changes in bedding and cage mate occlusions, which makes tracking multiple animals easier, but there is some loss of granularity. This means that top down systems are unable to detect fine motor movements such as grooming (Salem et al., 2015).

Another proven method of tracking is the use of radio frequency identifier (RFID) microchips (Rao and Edmondson, 1990, Dell’Omo et al., 1998, Calvez et al., 2006). Such systems track the identity and location of RFID-chipped animals as they can trigger multiple RFID readers at set locations (Lipp, 2005, Freund et al., 2013). IntelliCage (TSE systems GmbH) is one of the first fully automated testing apparatus that uses RFID reader coils to monitor behaviours in group housed RFID tagged mice (Lipp, 2005). Over the years the use of RFID readers has been developed for use with infrared sensors, weighing scales and lickometers to get a comprehensive data set from a large group of animals simultaneously without experimenter intervention (Krackow et al., 2010, Benner et al., 2015). This allows for a large number of animals to be tested simultaneously for long periods of time; however animals still need to be moved from their home cages into this bespoke environment for the testing period. Winter and Schaefers (2011) resolved this issue by adding an automated sorting system that allows individual animals to enter or leave a test arena from its home cage by triggering a gate with its RFID tag. Phenoworld (TSE systems) combined the above two approaches and offers bespoke testing environments, where the animals are housed in the central IntelliCage chamber and this is connected to various testing arenas through AnimalGate, all data collection is fully automated and experimenter intervention is almost negligible. There are also a number of non-commercial systems, such as the system developed by Weissbrod et al. (2013), that follow the same principle but are limited by the number of parameters they can record.

Even so such systems are incompatible with a standard mouse vivarium and require the mice to be removed into a bespoke environment for the duration of the experiment (See Table 1).

**Table 1:** Comparison of home cage monitoring systems.

System	Company/Institution	Camera Position	Strengths	Limitations	References
Home Cage Environment	CleverSys Inc	Side	Detailed assessment of animal behaviours in home cage.	Solitary housing. No direct measurement of activity	Steele et al. (2007) Roughan et al. (2009)
GroupHousedScan	CleverSys Inc	Top and Side	Detailed assessment of animal behaviours and social interactions in home cage.	Maximum of two individuals. Not compatible with standard vivarium	–
PhenoRack	ViewPoint Life Sciences	Side	Compatible with High density IVC racks. Quantitatively measures activity and Automatically annotates rearing, drinking and feeding in home cage.	Solitary housing.	Aniszewska et al. (2014)
SCORHE	NIH	Front and Rear	Compatible with standard high density IVC rack. Measures activity in home cage.	Solitary housing. Currently limited to black mice.	Salem et al. (2015)
PhenoTyper	Noldus Information Technology	Top	Can be multiplexed with drug delivery systems and operant systems to run bespoke experiments	Solitary housing in bespoke environment. Top view camera cannot track detailed behaviours.	de Visser et al. (2006) Spink et al. (2001)
ANY-Maze Cage	Global Biotech Inc; Stoelting Co.	Top	Can be used in combination with weight transducers for measuring food and water consumption. Can measure running wheel activity and immobility defined sleep	Solitary housing. Mice need to be removed to bespoke environment.	Fisher et al. (2012) Banks et al. (2015)
Home Cage Analysis System	Actual Analytics	Side	Mice remain in their home cage within established social groups. Social interactions Compatible with standard high. density IVC racks Videos overlaid with RFID identities for each mouse.	Takes two IVC rack spaces. RFID microchips inserted into mouse groin under general anaesthesia.	Bains et al. (2016)
LABORAS	Metris, B.V.	N/A	Registers behavioural signatures for fine movements like grooming, eating and drinking.	Solitary housing. No video output. Mice need to be removed into bespoke environment.	Quinn et al. (2003) Goulding et al. (2008)
IntelliCage	NewBehaviour TSE Systems	N/A	Group housing. Possible to design custom cages set ups to run bespoke experiments.	No video output therefore detailed behaviours not recorded. Animals need to be acclimatised to the bespoke environment.	Lipp (2005) Endo et al. (2012)
PhenoMaster	LabMaster TSE Systems	N/A	Multiplexed with weight transducers and beam break detectors for measuring food and water intake along with activity measurements. Indirect gas calorimetry. Can be multiplexed with other equipment to run memory and learning tasks.	Solitary housing. Mice need to be removed to bespoke environment	Clemmensen et al. (2015)

Our ability to record automated detailed behavioural parameters over time in an undisturbed cage encouraged us to explore whether true home cage phenotyping was feasible. True home cage in this context was defined as a normal rack-mounted cage, where mice are born, reared and housed within their established social groups (Bains et al., 2016). To evaluate the system we focused, first of all, on activity measurements over extended periods in inbred strains. Our findings (Fig. 1) showed that the system can discriminate between individual strain activity patterns over 24-h in the home cage. We found that activities are distributed throughout the 24-h period with animals being active through the first quarter of the light phase, showing anticipatory activity prior to the onset of the dark phase and a period of inactivity during the final third of the dark phase. Furthermore, we found that many of these behaviours were strain specific. In FVB mice, for example, we could not distinguish activities in the light and dark phases quite as readily as in C57BL/6 mice.

**Fig. 2:**
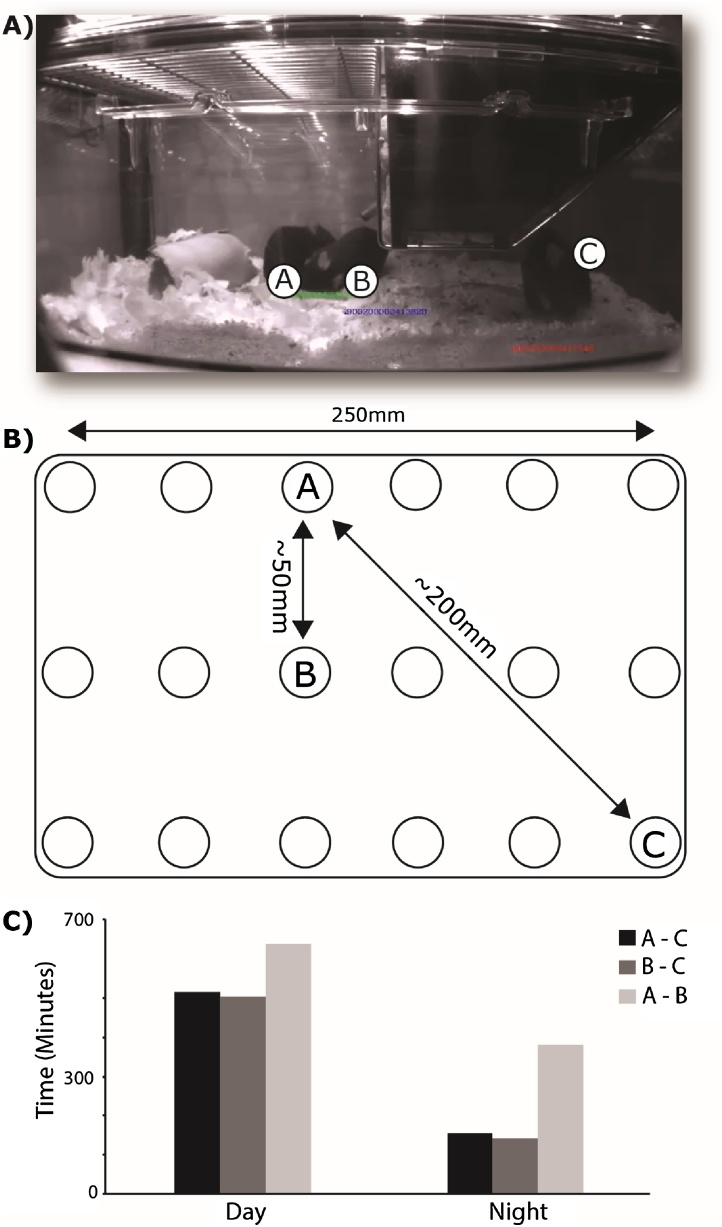
Home-cage social proximity interactions. RFID tracking of mice in multiple occupancy cages enables an estimate of social proximity scores. A) still from a video with overlay of RFIDs for individual animals; B) the base plate array recognises unique RFIDs and records animal locations concurrently; C) cumulative time spent in close proximity (<75 mm) during day and night for each pair of animals in the cage. In this instance, animals A and B spend less time interacting closely with animal C and this is more apparent at night.

Using the HCA system, we have been expanding our analysis to investigate behavioural interactions in a group-housed home-cage setting. Tracking interacting animals and monitoring social groups using a combination of detectors and video analysis is not new. Weissbrod et al. (2013) devised a system for social behavioural phenotyping in semi-natural environments. However, environmental enrichment is known to influence the behavioural phenotype of genetically similar mice (Freund et al., 2013); therefore the phenotype expressed by individuals tested in such bespoke environments may not be directly comparable with those housed in conventional cages. Robust changes in social interactions over the dark and light phase can be observed in the mouse home cage using the HCA system (Fig. 2), where cumulative time spent in close proximity (<75 mm) to other individual cage mates can be recorded over time. Preliminary data shows that there are noticeable changes in proximity scores for night time, when the mice are more active and day time, when they are likely to be sleeping and huddled together in the nest. The latter behaviour might also be an indicator of thermoregulatory/metabolic function. Furthermore, the HCA system can be used to look at social behaviours in cages consisting of animals of mixed genotype. In this particular case we have observed significant bouts of social isolation during both light and dark phases in one of a number of behavioural mutants being studied in our group (Fig. 2C). In this instance, animal C is a mutant whereas animals A and B are wild-type littermates.

**Fig. 3:**
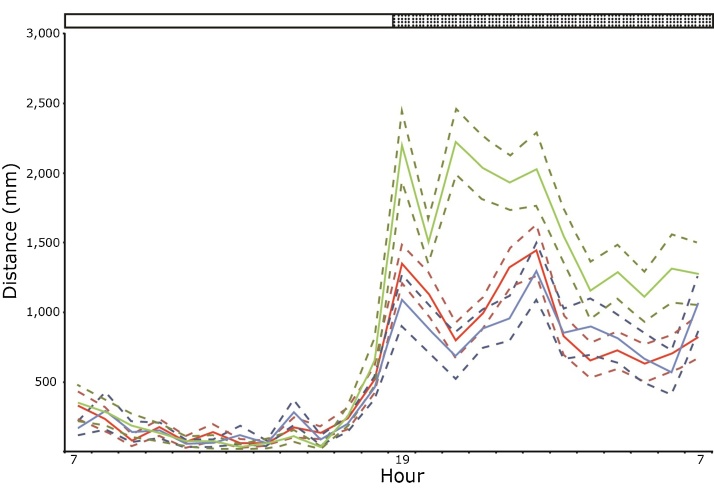
Nocturnal hyperactivity in a neurological mutant. Line plots illustrating the average hourly activity of 3 individual mice over a 24 h period. The solid lines represent the mean of the activity for each hourly period and the dotted lines represent the standard error of the mean activity over 3 consecutive days. The area under the shaded bar represents the dark phase while the area under the clear bar represents the light phase. The activity of all three animals within the cage is similar during the light phase but following lights-off the animal represented by the green line shows hyperactivity, which is sustained throughout the dark phase. (For interpretation of the references to colour in this figure legend, the reader is referred to the web version of this article.)

True home cage phenotyping over long periods has the potential to greatly enhance the study of a wide range of neurobiological diseases by enabling the accurate measurement of progressive behavioural changes in the same animals over weeks and months (Brooks et al., 2012). There is growing consensus that detailed understanding of behavioural changes in laboratory mice during sickness will help improve laboratory animal welfare by informing severity limits and humane endpoints (Littin et al., 2008; Wearly et al., 2009; Richardson, 2015). However building such a detailed picture using cage side assessment alone is difficult, as mice are most active during dusk and dawn periods, which means that behaviours that are most indicative of ill health go unobserved (Hawkins, 2002). Moreover mice are prey species, they are naturally inclined to hide any weaknesses from potential predators (Weary et al., 2009). Therefore, during cage-side checks, mice will often mask any behavioural indicators of ill health. However, we have observed that, as animals re-adjust to the IVC environment, significant changes in activity and sleep patterns occur, particularly during the dark phase. Fig. 3 shows activity data for a cage of three mutants with a progressive neurological deficit. While the three animals in the cage show similar activity during the light phase, one of the mutants shows sustained hyperactivity during the dark phase up until dawn. Without continuous monitoring over the light dark phase, it would not have been possible to observe this phenotype and its potential impact on the welfare of the animals would have gone unnoticed.

**Fig. 4:**
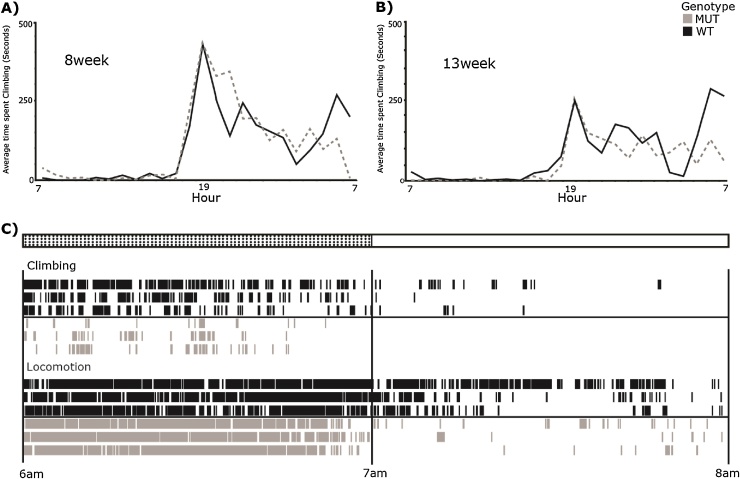
Automated assessment of home cage climbing activity in a mutant line with progressive motor deficits. Line graphs illustrating the average hourly climbing activities of 3 wild type (WT, black solid lines) and 3 mutant (MUT, grey dashed lines) mice. Climbing was assessed automatically using the HCA system. Average time spent climbing is noticeably higher in mice at 8 weeks of age A) than at 13 weeks of age B). Climbing behaviours in mutants appear to be significantly lower towards the end of the dark phase (6–7 a.m.) at both time points. C) Ethogram showing both locomotor activity and climbing activity in 13 week old mice over a two-hour period either side of lights-on (6 a.m.–8 a.m.). Mutant mice with motor deficits show particularly lower bouts of climbing activity at this time of day.

The combined use of RFID and infrared video monitoring allows one to track individual home-cage behaviours continuously. However, a remaining challenge is to use computer automation to record home-cage behaviours automatically, developed using previously-annotated video segments. The HCA system has the potential to record and automate a spectrum of biologically-relevant home-cage behaviours. As a preliminary study, we focused on the automated analysis of cage-bar climbing activity as a subtle measure of motor function in an unprovoked environment. Using this tool, climbing is detected over 6 frame blocks using the temporally smoothed output of a linear SVM classifier, trained (Fan et al., 2008) on over 7 h of annotated video footage (including over 130 separate bouts of climbing) using a Local Trinary Pattern representation (Yeffet and Wolf, 2009) of the upper portion of the cage. We used this automated system to analyse climbing activity in detail over 3 consecutive days in a mouse line with progressive motor deficits (MUT) with wild type littermate controls (WT) at 8 and 13 weeks of age (Fig. 4). Preliminary data indicates that a specific time-dependent decrease in climbing activity, detected using the automated system, is a strong indicator of disease onset in this line.

## 7 Concluding comments

Most behavioural phenotyping relies on the use of multiple testing platforms to study any one behavioural domain. For example, motor function or anxiety in mice can be tested using numerous tests while each can provide different, but informative, phenotypic information. Likewise, the techniques described in this review have the potential to open a whole new dimension of mouse behaviour by shedding light on aspects of mouse behaviour that have hitherto gone unobserved. Moreover, rather than challenging the validity of existing work, these techniques should provide complementary information in establishing complex behavioural phenotypic patterns in mice. For example, while group-housed home-cage behaviours can provide valuable information on multiple social behaviours, social entrainment cues themselves might negatively impact upon the innate circadian period or sleep/wake behaviour of an individual animal. In these instances, amongst others, it would still be preferable to study the behaviour in singly housed conditions where the impact of social interaction will not mask the behaviour of the individual.

Our findings highlight the importance of testing mouse behaviours over extended periods in undisturbed conditions (for at least one 24-h cycle). The discriminatory power of these tests is far greater than conventional out-of-cage phenotyping. However, testing during the dark phase need not necessarily be the most informative or most discriminative as test outcomes are dependent on the specific behaviours or on the particular mouse strain/mutant line being studied. Careful consideration should be given to individual behavioural studies but, ultimately, longitudinal studies throughout the light:dark cycle are desirable. Progress with the automation and diversity of home cage testing will be a critical factor in enabling such studies. Though the field has seen an explosion of several commercial and non-commercial systems, the challenge ahead lies in the ensuring that these systems are robustly validated through manual and cross laboratory validation.

## Conflict of interest statement

The authors RS and JA were/are employed by or were shareholders in Actual Analytics Ltd at the time the research was performed and therefore declare a competing financial interest. Actual HCA is commercially available from Actual Analytics Ltd.
